# Cuticle Protein LmACP19 Is Required for the Stability of Epidermal Cells in Wing Development and Morphogenesis of *Locusta migratoria*

**DOI:** 10.3390/ijms23063106

**Published:** 2022-03-13

**Authors:** Xiaoming Zhao, Ti Shao, Yazhi Su, Jing Zhang, Xin Gou, Weimin Liu, Jianzhen Zhang

**Affiliations:** 1 Institute of Applied Biology, Shanxi University, Taiyuan 030006, China; st18584554205@163.com (T.S.); syz18834850773@163.com (Y.S.); ZJ15235142240@163.com (J.Z.); ychunlingzxm@sina.com (X.G.); liweimin@sxu.edu.cn (W.L.); 2 College of Life Science, Shanxi University, Taiyuan 030006, China

**Keywords:** cuticle protein, wing development, wing morphogenesis, *Locusta migratoria*

## Abstract

Insect wing consists of a double layer of epidermal cells that produce and secrete the dorsal and ventral cuticular components. It is important for the stability of epidermal cells during wing development and morphogenesis, but its specific gene expression and physiological function during this process remain unclear. In our previous work, a wing cuticle protein gene *LmACP19* was identified in *Locusta migratoria* based on transcriptomic data. Here, we report on its roles in wing development and morphogenesis. *LmACP19* encodes a chitin-binding protein belonging to RR-2 subfamily of CPR family, which is highly homologous to CP19-like proteins in other insect species. RT-qPCR analysis revealed that *LmACP19* is highly expressed in wing pads of fifth-instar nymphs, and its encoded protein is located in two layers of epidermal cells but not in the cuticle. Suppression of *LmACP19* by RNA interference led to abnormal wing pad and wing morphogenesis with curved, unclosed, and wrinkled phenotypes during nymph-to-nymph and nymph-to-adult transition, respectively. Furthermore, deficiency of *LmACP19* affected arrangement of epidermal cells, resulting in apoptosis. Our results indicate that *LmACP19* is indispensable for wing development and normal morphological structure by maintaining the stability of epidermal cells during *L. migratoria* molting.

## 1. Introduction

Insects are the largest class of the phylum Arthropoda, which are characterized by their exoskeleton structure, segmented body, and paired and jointed appendages. The exoskeleton of insects is formed by layered cuticles that mainly consist of chitin and associated proteins, which are produced by epidermal cells. Insect cuticle is composed of three functionally distinct horizontal layers, including the outermost waterproof envelope, the middle protein-enriched epicuticle, and the innermost chitinous procuticle that contacts the apical plasma membrane of epidermal cells [1]. The procuticle can be further divided into exocuticle and endocuticle that interfaces with the epidermis. In general, the exocuticle is formed before insect ecdysis, whereas the endocuticle begins to deposit after ecdysis and ends just before the next molting [2]. Because the nongrowth of cuticle restricts the growth and development of insects, the cuticle must be periodically replaced and remodeled during molting. The epidermal cells play a crucial role in the molting process, which produce and release a variety of enzymes that degrade the endocuticle of old cuticle, and then synthesize a new cuticle [3].

Wings are important appendages for insects and an indispensable structure in many insect species. They develop internally as larval organs in the body of holometabolous insects, or form from the wing pads of the nymph in hemimetabolous insects [3]. Wings play important roles in foraging, courtship, protection against predators, and migratory flight. Different from single-layer epidermal cells of the exoskeleton, insect wings from adult or wing pads from nymph are made up of two layers of epidermal cells that underlie the dorsal and ventral cuticle [4]. The double-layered cell structure of wings is formed by living epidermal cells as a close arrangement, and this tight arrangement in the wings is controlled by a variety of cell adhesion genes [5]. Wing morphology is not only defined by the cuticle, but also by the underlying epidermal cells. During the development of epidermis, cell junctions play a crucial role in the correct organization and function of the entire tissue [6].

Cuticular proteins (CPs) and chitin are the main structural components of the exocuticle and endocuticle layers that comprise the procuticle. During insect molting, CPs are periodically synthesized [7]. In insect genomes, a large number of genes were identified that encode CPs or CP-like proteins. To date, more than 200 putative CP or CP-like genes have been identified in *Drosophila melanogaster* [8], *Bombyx mori* [9], *Anopheles gambiae* [10], and *Mandula sexta* [11], and 13 different families of their encoding proteins were defined by their unique amino acid sequence motifs [12], including CPR family, CPAPs family, CPT family, CPG family, etc. The CPR family is the largest CP family containing a Rebers & Riddiford consensus (R&R consensus) of 44 amino acid residues [13], which was divided into three subfamilies according to the R&R consensus, namely RR-1, RR-2, and RR-3. As reported previously, CPs or CP-like proteins of the CPR family are the main components of insect exoskeleton and determine the different physical properties of the cuticle at different developmental stages, as well as different body regions by interacting with chitin or other CPs [14]. For instance, in the silkworm *B. mori*, a dysfunctional RR-1 protein encoded by *BmCPR2* in a *stony* mutant results in dramatically reduced chitin content in cuticle, limitation of cuticle extension, and abnormal distribution of internode and intersegmental fold in larvae [15]. In the red flour beetle *Tribolium castaneum*, RNAi targeting two major structural RR-2 protein genes, *TcCPR27* and *TcCPR18*, caused disorder of the laminar structure, resulting in wrinkled and improperly expanded elytra [16]. The migratory locust *Locusta migratoria*, a heterometabolous insect, is an orthopteran migratory pest, which has a strong flight capability dependent on its powerful fore- and hindwings. In *L. migratoria*, 51 CPRs were identified based on our previous transcriptomic data [17]. However, their function and mechanism in the formation and development of the wing cuticle remain unclear.

In our previous work, three sequences of wing cuticle protein genes, *LmACP7*, *LmACP8*, and *LmACP19*, were identified and further confirmed in the transcriptome of *L. migratoria* [17]. We subsequently found that all the three cuticle proteins encoded by these genes belong to RR-2 subfamily of the CPR family, which might be associated with rigid (hard) cuticles [18]. Among them, LmACP7 is an indispensable structural component needed for wing morphological and structural integrity of wing cuticle in the migratory locust [19]. *LmACP8* is negatively regulated by the hormone receptor LmHR39-mediated 20-hydroxyecdysone signaling pathway and is involved in wing development during nymph to adult transition [20]. It is noteworthy that LmACP7 accumulates in the exocuticle, while LmACP8 is located in the exocuticle and endocuticle of wing pads and adult wings and co-locates with chitin. Both of them are required for normal wing morphogenesis as an important structural component in the wing cuticle. In the present study, we found LmACP19 mainly existed in two layers of epidermal cells during the wing development. We further demonstrated that deficiency of *LmACP19* affected arrangement of epidermal cells and resulted in apoptosis. Our results indicate that *LmACP19* is necessary for maintaining the stability of epidermal cells and wing morphological structure in *L. migratoria*.

## 2. Results

### 2.1. Identification and Characterization of LmACP19

**Figure 1:**
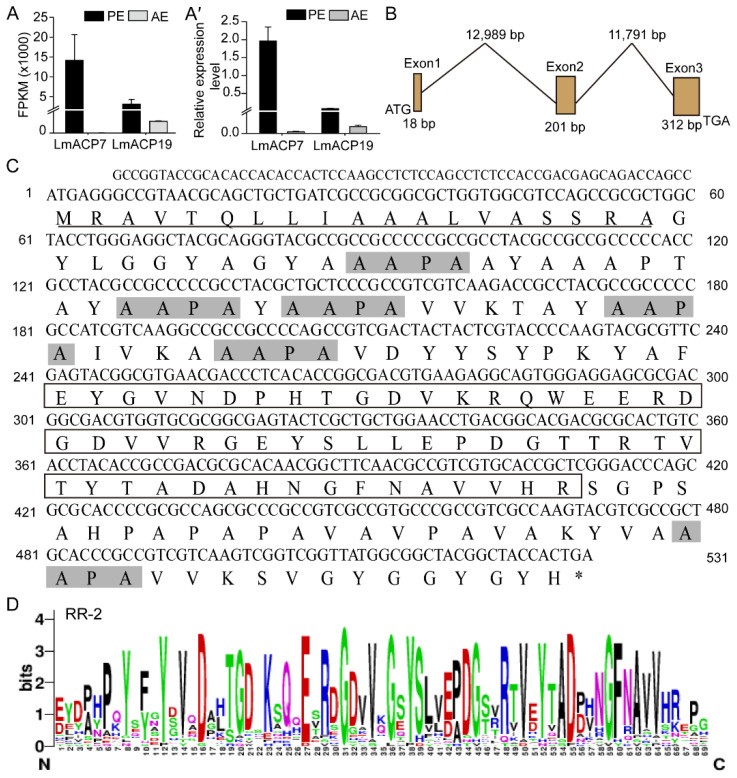
Identification and bioinformatics analysis of LmACP19 in *Locusta migratoria*. (**A**) Wing cuticle protein genes were identified based on the transcriptome of locust wing. (**A′**) Wing cuticle protein genes were determined at pre-ecdysis and after ecdysis by RT-qPCR. PE: pre-ecdysis; AE: after ecdysis. (**B**) *LmACP19* gene structure. (**C**) The sequence of LmACP19. Underline: signal peptide; the box: RR-2 motif; the gray shadow: AAPA motifs. (**D**) WebLogo analysis of RR-2 motifs in insect cuticular proteins. RR-2 motifs were obtained from 26 CPR family (RR-2 subgroup) proteins, including LmACP19. WebLogo online sever was used to identify the abundance of each amino acid residue within the consensus sequence of RR-2 proteins.

Based on our previous comparative transcriptomics [17,19], two wing cuticle protein genes (*LmACP7* and *LmACP19*) were identified, which were highly expressed in pre-ecdysis (Figure 1A–A′). We previously demonstrated that *LmACP7* was involved in the development of wings. In this study, we further explore the function of *LmACP19* during the development of locust wing. We firstly obtained the cDNA sequence of *LmACP19* and assembled its intron/exon organization, and found that the *LmACP19* was made up of three exons with an open reading frame of 531 bp (Figure 1B). The *LmACP19* encodes a protein with 176 amino acid residues containing a putative signal peptide sequence (amino acids 1–19) and an RR-2 motif (amino acids 81–136) with a theoretical molecular mass and pI of 18.08 kDa and pKa 7.88, respectively (Figure 1C,D).

The orthologues of LmACP19 from other insect species were obtained by BLAST in NCBI, and the predicted structure of all these proteins contains a signal peptide and chitin binding domain (Supplementary Figure S1A). Phylogenetic analysis using amino acid sequences from other insect species indicates that these proteins could be divided into two clades, CP19 and CP19-like, and the LmACP19 was clustered with CP19-like and had a high relationship with that from Blattaria (Supplementary Figure S1B). Multi-sequence alignment analysis showed that CP19 and CP19-like proteins contained several conserved motifs (AAPA) in addition to signal peptides and RR-2 motifs (Supplementary Figure S1C).

### 2.2. LmACP19 Is Mainly Expressed in Wing Pad Cells

**Figure 2:**
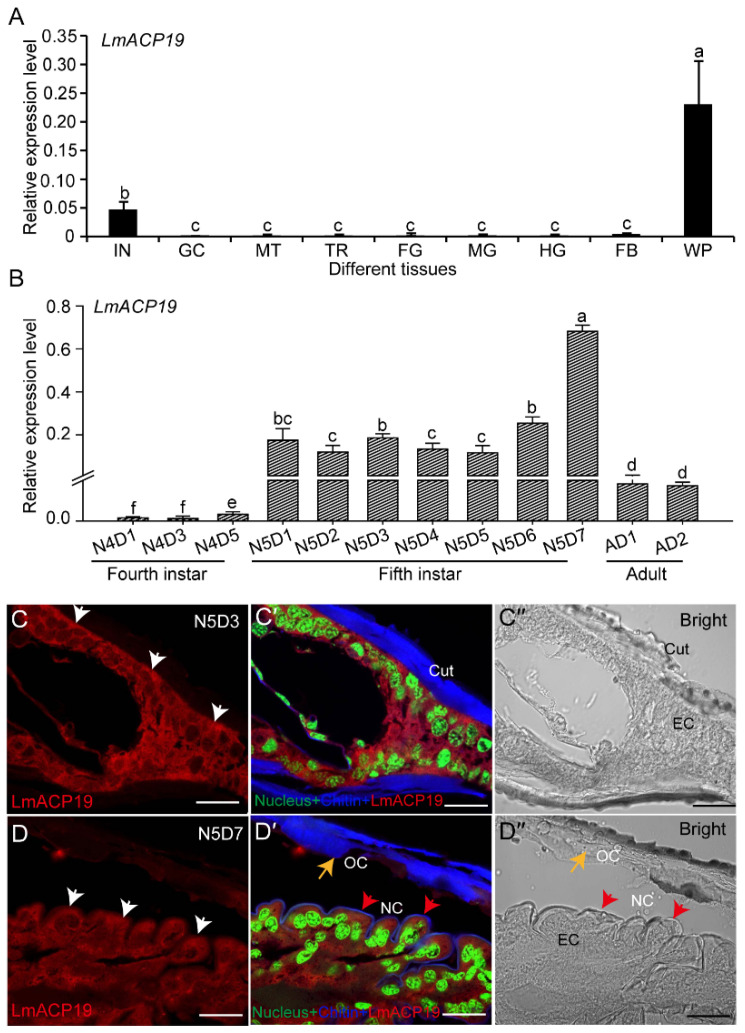
Expression of *LmACP19* in different tissues and developmental stages and localization of LmACP19. (**A**) Expression of *LmACP19* in different tissues of fifth-instar nymphs as detected by RT-qPCR. Different tissues are listed: IN, integument; GC, gastric caeca; MT, Malpighian tubules; TR: trachea; FG, foregut; MG, midgut; HG, hindgut; FB, fat body; WP, wing pads. (**B**) Expression of *LmACP19* in different stages (from fourth-instar nymph to adult) as detected by RT-qPCR. *RPL32* was used as the reference control. All data are reported as means ±SE of four independent biological replications. Different letters (a–f) on the bars indicate significant difference among different samples (*p* < 0.05, Tukey’s HSD test, *n* = 4). (**C**–**C″** and **D**–**D″**) Localization of LmACP19 in middle- and late-stage wing pads of fifth-instar nymphs by immunochemistry, scale bar = 20 μm; SYTOXR Green nucleic acid stain: nucleus; FB28: chitin; LmACP19: anti-LmACP19. OC: old cuticle, NC: new cuticle; white arrow indicates LmACP19 signal in cytoplasm, red arrow indicates new cuticle, yellow arrow indicates old cuticle.

To explore the function of *LmACP19*, we determined its tissue-specific expression pattern in fifth-instar nymphs by RT-qPCR analysis. The results showed that *LmACP19* was highly expressed in the wing pads from N5D6 nymphs of *L. migratoria*, followed by integument, but showed low expression levels in other tested tissues (Figure 2A). Analysis of the developmental expression pattern showed that *LmACP19* had the most abundant transcripts in the wing pads of fifth-instar nymphs, and had high mRNA expression levels before molting in each instar nymphs (N4D5 and N5D7), whereas low levels were noted early in each stage (N4D1–N4D3 and N5D1–N5D5), and transcript levels were stabilized in adult wings (day 1 and day 2) (Figure 2B).

To analyze the localization of LmACP19, paraffin sections of wing pads at the middle and late stages of fifth-instar nymphs were prepared for immunohistochemistry experiments. Immunohistochemistry analysis showed that the signal of LmACP19 was mainly detected in epidermal cells of wing pads at the middle and late stages, but not co-localized with chitin in the procuticle of wing pads (Figure 2C–C″ and D–D″), suggesting that LmACP19 mainly existed in the epidermal cells of wing pads in fifth-instar nymphs, but not in the procuticle.

### 2.3. LmACP19 Is Required for the Wing Morphogenesis

**Figure 3:**
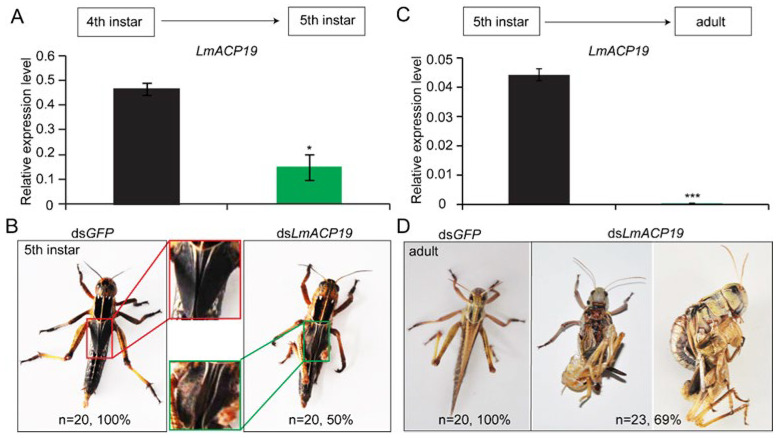
The phenotype of wing pads and wings after suppressing *LmACP19.* (**A**) Expression of *LmACP19* after RNAi in fourth-instar nymphs as detected by RT-qPCR. *RPL32* was used as the reference control. Data are reported as means ± SE of four independent biological replications; asterisks indicate significant differences, *, *p* < 0.05. (**B**) The phenotype of wing pads after suppressing *LmACP19* in fourth-instar nymphs. (**C**) Expression of *LmACP19* after RNAi in fifth-instar nymphs as detected by RT-qPCR. *RPL32* was used as the reference control. Data are reported as means ±SE of four independent biological replications; asterisks indicate significant differences, ***, *p* < 0.001. (**D**) The phenotype of wings after suppressing *LmACP19* in fifth-instar nymphs.

To investigate the biological role of *LmACP19* during nymph–nymph and nymph–adult development of *L. migratoria*, RNAi was performed by injecting dsRNA into fourth-instar nymphs and fifth-instar nymphs, respectively. After injecting dsRNA, the *LmACP19* transcript level was suppressed in the wing pads by 67% and 97% compared with the control, as determined by RT-qPCR, in the fourth-instar nymphs and fifth-instar nymphs, respectively (Figure 3A,C). The results showed that deficiency of *LmACP19* in the fourth-instar nymphs led to the abnormal wing pads that exhibited curved and unclosed phenotype (in about 50% of injected nymphs) during nymph–nymph transition (Figure 3B). In particular, about 69% of the adult wings were wrinkled and were not shed during nymph–adult transition after injecting ds*LmACP19* into fifth-instar nymphs (Figure 3D). In contrast, no visible changes in phenotypes were observed in the control group injected with ds*GFP* during nymph–nymph and nymph–adult transition, respectively (Figure 3B,D). These results indicate that *LmACP19* is required for the wing morphogenesis during molting.

### 2.4. Deficiency of LmACP19 Affected the Arrangement of Epidermal Cells

**Figure 4:**
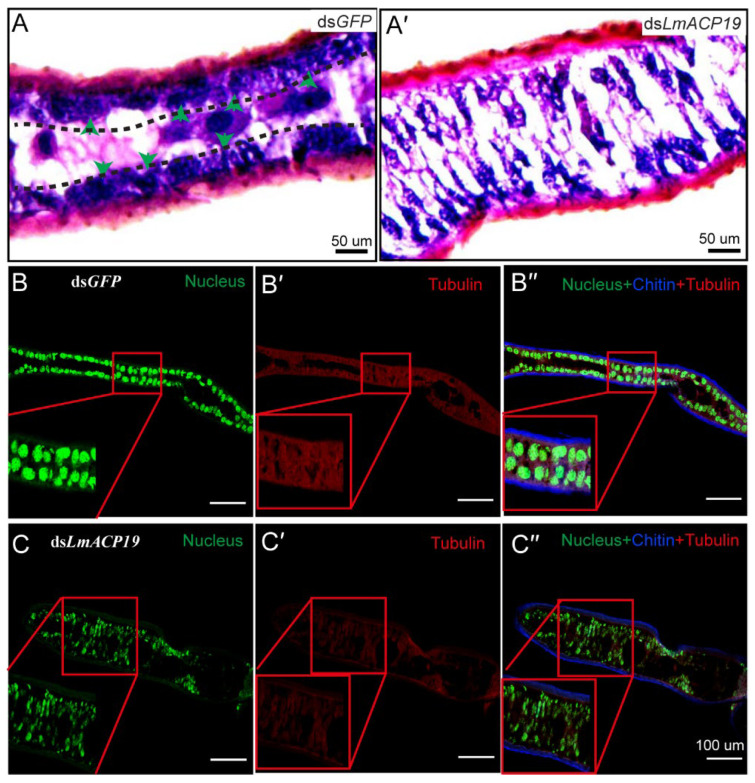
Effect of ds*LmACP19* RNAi on the cytoskeleton of wing pads of *L. migratoria.* (**A**–**A′**) The microstructure of wing pads of fifth-instar nymphs was observed after RNAi with ds*LmACP19* or ds*GFP* at day 2 of fourth-instar nymphs by H&E staining. Scale bar = 50 μm. (**B**–**B″** and **C**–**C″**) The cytoskeleton of wing pads observed after RNAi by immunohistochemistry. SYTOXR Green nucleic acid stain: nucleus; FB28: chitin; Tubulin: anti-tubulin. Scale bar = 100 μm.

To explain the abnormal morphology of wing pads during nymph–nymph transition, microsections of the fixed and embedded wing pads were prepared. H&E staining revealed that the arrangement of epidermal cells between the ventral and dorsal cuticular layer from the ds*LmACP19*-treated insects was disordered compared with the ds*GFP*-treated insects (Figure 4A–A′). To confirm the morphological changes of the wing pads after silencing *LmACP19*, the cytoskeleton was also stained with tubulin antibody and SYTOX R Green nucleic acid stain. Compared to the ds*GFP*-treated insects, we clearly observed disorganized and abnormally arranged epidermal cells in the ds*LmACP19*-treated insects (Figure 4B–B′,C–C′), suggesting that *LmACP19* is required for the stability of epidermal cells.

**Figure 5:**
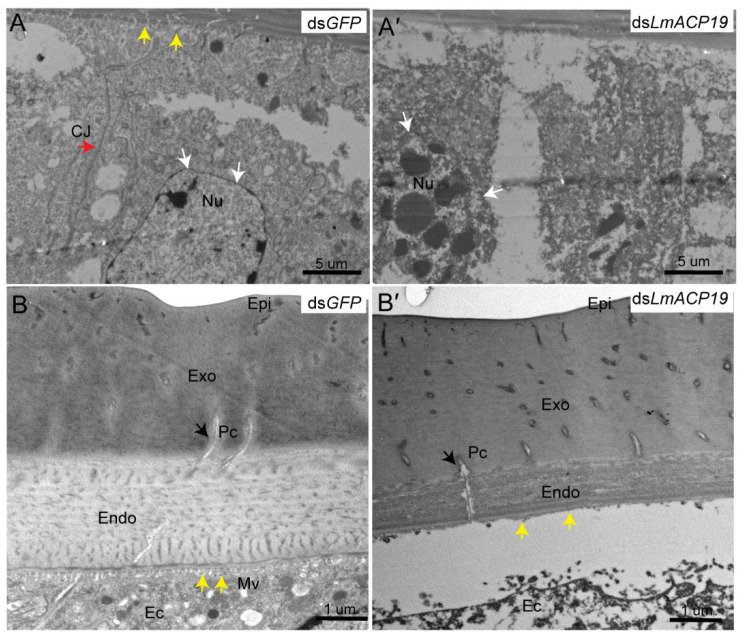
Effect of ds*LmACP19* RNAi on structure of adult wings of *L. migratoria.* (**A**–**A′**) The structure of epidermal cells was observed after RNAi in fifth-instar nymphs through TEM. Scale bar = 5 μm. (**B**–**B′**) The structure of wing cuticle was observed after RNAi in fifth-instar nymphs through TEM. Epi: epicuticle, Endo: endocuticle, Exo: exocuticle, Ec: epidermal cell, Pc: pore canal, Nu: nucleus, Mv: microvilli, CJ: cell junction. Scale bar = 1 μm. Black arrows indicate pore canal, white arrows indicate nucleus, yellow arrows indicate microvilli, red arrows indicate cell junction.

To further confirm this result, the structure of wing epidermal cells was observed by TEM. The results showed that the apical plasma membranes of epidermal cells in wings form short microvilli, and that there are visible cell junctions by which epidermal cells are held together in ds*GFP*-treated control insects (Figure 5A). However, after injection of ds*LmACP19*, the epidermal cells of locust wings were fragmented, and both microvilli and cell junctions were degraded to varying degrees (Figure 5A′). Meanwhile, morphological features of cell death, such as cell pyknosis and nuclear fragmentation, could be clearly observed (Figure 5A′). As a control, the epidermal cells of integument were normal in both ds*GFP*-treated control insects and ds*LmACP19*-injected insects (Supplementary Figure S2A–A′).

Furthermore, the results of TEM analysis also showed that the endocuticle of wing treated with ds*LmACP19* in fifth-instar nymphs was noticeably thinner than that of the ds*GFP*-treated control, and the lamellae in the region connecting the endocuticle and the apical plasma membrane of epidermal cells were disrupted, and the microvilli were degraded to different extents (Figure 5B–B′). As a comparison, the cuticle structure of integument treated with either ds*GFP* or ds*LmACP19* remained unaltered (Supplementary Figure S2B–B′). Together, these results indicate that *LmACP19* is needed for the arrangement of wing epidermal cells during molting.

### 2.5. Deficiency of LmACP19 Results in Apoptosis of Epidermal Cells

**Figure 6:**
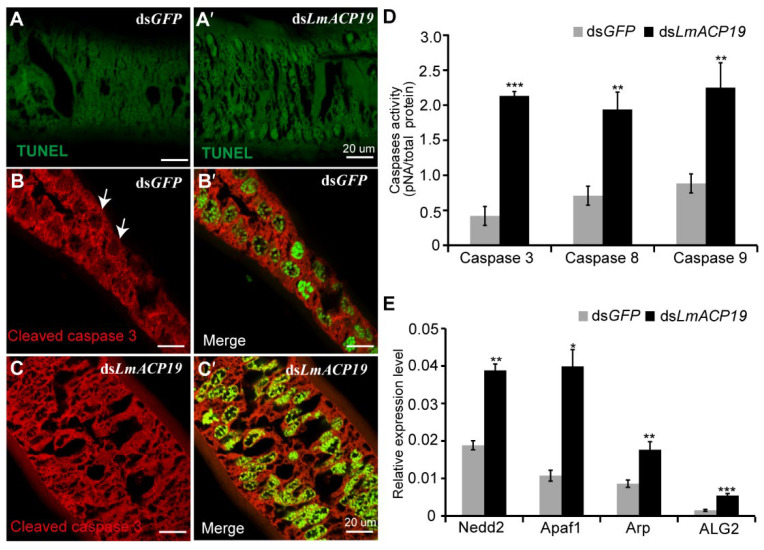
Deficiency of *LmACP19* induces apoptosis response in wing pads after ecdysis. (**A**–**A′**) TUNEL staining was performed after RNAi with ds*LmACP19* or ds*GFP* at day 2 of fourth-instar nymphs. Scale bar = 20 μm. (**B**–**B**′ and **C**–**C′**) Cleaved caspase-3 was detected by immunohistochemistry with the antibody to cleaved caspase-3 after RNAi with ds*LmACP19* or ds*GFP* at day 2 of fourth-instar nymphs. (**D**) The caspase-3, caspase-8, and caspase-9 activities were determined using caspase activity kit, respectively. Data are reported as means ±SE of three independent biological replications; asterisks indicate significant differences, **, *p* < 0.01, ***, *p* < 0.001. (**E**) Apoptosis-related genes were detected by RT-qPCR. *RPL32* was used as the reference control. Data are reported as means ± SE of three independent biological replications; asterisks indicate significant differences, *, *p* < 0.05, **, *p* < 0.01, ***, *p* < 0.001.

According to the results above, we speculated that deficiency of *LmACP19* could induce apoptosis during molting because of the disorganization of the epidermal cells. To test this hypothesis, TUNEL staining analysis was first performed using wing pads of fifth-instar nymphs after silencing *LmACP19*. The results showed that, compared to the ds*GFP*-injected nymphs, an apoptosis-like process was detected in ds*LmACP19*-injected nymphs (Figure 6A–A′). As revealed by immunohistochemistry with an antibody against cleaved caspase 3 (an apoptotic effector), the cleaved caspase-3 was detected in the nuclei of epidermal cells of ds*LmACP19*-injected nymphs, but only in the cytoplasm of ds*GFP*-injected control nymphs (Figure 6B–B′ and C–C′). We subsequently assayed caspase activities, markers of apoptosis. As shown in Figure 6D, the caspase-3, caspase-8, and caspase-9 activities were significantly increased in ds*LmACP19*-injected nymphs compared with ds*GFP*-treated control insects. Furthermore, the mRNA expression level of apoptosis regulator, apoptotic protease-activating factor, and initiator genes (*Nedd2*, *Apaf1*, *Arp*, and *ALG2*) were also significantly increased in the ds*LmACP19*-injected locusts compared to the ds*GFP*-injected locusts (Figure 6E). These results support the hypothesis that a decrease in *LmACP19* expression results in apoptosis of epidermal cells during molting.

## 3. Discussion

In this work, we characterized a cuticular protein gene *LmACP19* based on transcriptomic data of wing pads, which encodes a protein belonging to subgroup RR-2 of the CPR family. The transcripts for this gene are expressed primarily in the wing pads of *L. migratoria*. The RR-2 proteins are primarily involved in the formation of rigid and sclerotized cuticle, such as the elytra cuticle in *T. castaneum* [2,21]. Thus, we hypothesized that *LmACP19* may be related to the development of wing cuticle and morphogenesis in *L. migratoria*.

The time period of CP gene expression defines the type of CPs that constructs the layer and determines the physical property of each cuticular layer during insect molting [2]. According to previous studies, a large number of CP genes have been identified at different developmental stages and cuticle-associated parts of insects. For example, four CP genes encoding post-ecdysial proteins were characterized from locust nymph cuticle and adult cuticle, respectively [22,23], whereas 20 CP genes encoding pre-ecdysial proteins were found in the pharate adult cuticle of *L. migratoria* [24,25,26]. These CPs may be involved in the formation of cuticle at different stages. In wing discs of *B. mori*, 52 CP genes were identified and detected for their period expression profiles. Among them, the CP genes encoding the RR-2 proteins have a peak expression before pupation, whereas the CP genes that encode RR-1 proteins are highly expressed before and after pupation [27]. Subsequently, TEM immunogold detection indicated that RR-1 proteins were localized in the procuticle of the soft intersegmental membrane, while the proteins of RR-2 subgroup consistently existed in hard cuticle and not in flexible cuticle in *A. gambiae* [28]. These previous reports suggest that CP genes encoding RR-1 or RR-2 proteins expressed at certain times might be involved in the construction of specific cuticular layers. In this study, we found that *LmACP19* was mainly expressed in the pre-ecdysis based on our transcriptome data and RT-qPCR analysis (Figure 1A–A′ and Figure 2B), which is similar to that of the other two wing cuticle protein genes *LmACP7* and *LmACP8* that also encoded a member of subgroup RR-2 of the CPR family. We previously confirmed that LmACP7 protein accumulates in the exocuticle, while LmACP8 is located in the exocuticle and endocuticle of wing pads and adult wings [19,20]. Compared to LmACP7 and LmACP8, immunohistochemistry revealed that LmACP19 only was expressed in the epidermal cells of wing pads during wing development (Figure 2C), implying that LmACP19 may play different physiological functions in wing cuticle development than LmACP7 and LmACP8. In our previous work, suppressing *LmACP7* or *LmACP8* expression by RNAi only resulted in wing defects during nymph–adult transition, and the resulting adult wings were not fully elongated but wrinkled [19,20]. Here, injection of ds*LmACP19* into fourth- and fifth-instar nymphs resulted in wing pads and wing defects during nymph–nymph transition and nymph–adult transition, respectively. In particular, the wing pads were curved, leading to wrinkled wings in the ds*LmACP19*-treated insects (Figure 3). These phenotypes after silencing *LmACP19* are different from those with suppression of *LmACP7* or *LmACP8*, which may depend more on location and properties of individual CP proteins.

We previously demonstrated that dysfunction of *LmACP7* or *LmACP8* resulted in disordered lamellae of the exocuticle and the thickness of the endocuticle was dramatically decreased during wing development [19,20]. Similarly, we also found that the thickness of the endocuticle was dramatically decreased during wing development after suppressing *LmACP19* from the ultrastructure level, but not the exocuticle (Figure 5), suggesting that *LmACP19* is required for structural integrity of the wing endocuticle during molting. However, H&E and cytoskeleton staining revealed that the epidermal cells of wing pads in the ds*LmACP19*-treated insects were disorganized and arranged abnormally compared with the ds*GFP*-treated insects (Figure 4B–B′ and C–C′), indicating that *LmACP19* is required for the stability of epidermal cells during wing development. In *D. melanogaster*, the integrity of the epidermis is required for embryonic development and organogenesis, and cells in the ventral epidermis of embryos lose adhesion and fall out of the epidermis, resulting in apoptosis [6]. Similarly, a novel cuticular protein gene, *BmorCPH24*, was required for the integrity of endocuticle and its disruption-induced apoptosis of epidermal cells in the silkworm *B. mori* [29]. In our previous work, we also found the epidermal cells of wings from ds*LmACP7*- or ds*LmACP8*-injected insects exhibited cell debris and loss of cell connections, and demonstrated that deficiency in *LmACP7* induced a cell apoptosis-like process [19]. In the present study, we found the integrity of epidermal cells was disrupted and the microvilli were degraded after suppression of *LmACP19* by RNAi. We also observed similar cell morphology to that of silencing *LmACP7* in the ds*LmACP19*-injected insects (Figure 5), which implies the suppression of *LmACP19* may induce cell apoptosis. To further test this possibility, TUNEL staining and immunohistochemistry analysis of cleaved caspase-3 indicate that an apoptosis-like process was involved because cleaved caspase-3 was detected in the nuclei of epidermal cells of ds*LmACP19*-injected nymphs. Furthermore, caspase activities (caspase-3, caspase-8, and caspase-9) and the mRNA expression level of apoptosis-related genes (*Nedd2*, *Apaf1*, *Arp*, and *ALG2*) were significantly increased in ds*LmACP19*-injected nymphs (Figure 6D,E). Taken together, our data support the hypothesis that a decrease in *LmACP19* expression results in the disordering of epidermal cells, leading to the induction of apoptosis during wing development.

Based on our previous work and current studies, we verified that these three wing cuticle protein genes, *LmACP7*, *LmACP8*, and *LmACP19*, play an important role in the formation and development of wing cuticle that is required for wing morphogenesis during molting. Our results provide insights into the molecular mechanism of how structural proteins in the flyable wing cuticle synergistically affect wing morphogenesis, but also highlight new and specific target genes for migratory pest control.

## 4. Materials and Methods

### 4.1. Insects

The eggs of *L. migratoria* stored in our laboratory were incubated and reared at 30 ± 2 °C, with a 14:10 h light: dark photoperiod, and 40 ± 10% relative humidity (RH) in a growth chamber. The newly hatched locusts were fed with fresh wheat sprouts, and wheat bran was supplied after nymphs reached the third instar. The fourth- and fifth-instar nymphs were used for RNA interference and total RNA isolation.

### 4.2. Bioinformatics Analysis

According to the transcriptomic data from nymphal wing discs [30] and our previous transcriptome data from the locust wing pads [19], the cDNA sequence of wing cuticle protein gene *LmACP19* was searched and identified. The translation of cDNA sequence was performed by ExPASy tools (http://www.expasy.org/tools/dna.html, accessed on 12 January 2022). The deduced protein domain was determined by SMART (http://smart.embl.de/, accessed on 10 January 2022), and GENEDOC software was used to perform multiple amino acid sequence alignments. Predictions of signal peptide, molecular mass, and isoelectric point (PI) of LmACP19 were analyzed using the EXPASY proteomics server (http://www.expasy.org, accessed on 14 January 2022). The common elements in the R&R consensus (RR-2) proteins were identified using WebLogo online sever (http://weblogo.berkeley.edu/logo.cgi, accessed on 14 January 2022). LmACP19 and its orthologous sequences were used to construct a phylogenetic tree by MEGA 6.0 software through the neighbor-joining method with 1000 bootstrap replicates. The GenBank accession numbers of LmACP19 and its orthologous sequences are listed in Supplementary Table S1.

### 4.3. Tissue-Specific and Developmental Expression Analysis

For tissue-specific expression analysis, nine different tissues, including the integument (IN), gastric caeca (GC), Malpighian tubules (MT), trachea (TR), foregut (FG), midgut (MG), hindgut (HG), fat body (FB), and wing pads (WP), were collected from the 6-day-old fifth-instar nymphs (N5D6). For analysis of the developmental expression, samples were collected from wing pads of fourth-instar nymphs (day 1, day 3, and day 5), fifth-instar nymphs (from day 1 to day 7), and wings after adult emergence (day 1 and day 2). All samples were collected with four biological replicates, each with four nymphs or adults. Total RNA of each sample was extracted using RNAiso™Plus (Takara Bio, Kusatsu, Japan) according to the protocol, and their quality and quantity were detected by 1.5% agarose gels and NanoDrop 2000 (Thermo Fisher Scientific, Waltham, MA, USA). The first-strand cDNA of each sample was synthesized by M-MLV reverse transcriptase (Takara Bio, Kusatsu, Japan) using 1 μg of total RNA as a template. The reverse-transcription quantitative polymerase chain reaction (RT-qPCR) was performed for the tissue-specific and developmental expression analysis as described previously [31]. The relative mRNA level of *LmACP19* was normalized to the expression of the internal marker gene, *RPL32*, which is stably expressed at all stages and in all tissues [32]. The primers used are listed in Supplementary Table S2.

### 4.4. Antibody Preparation and Immunohistochemistry

The coding region of *LmACP19* was amplified using specific primers with a BamHI/NotI site (Supplementary Table S2). The purified PCR product digested with BamHI/NotI was cloned into pET32a vector (Novagen, Germany) to construct the recombinant expression vector pET32a–LmACP19. Positive clones were selected and transformed into *Escherichia coli* strain BL21 (DE3) cells (TransGen, Beijing, China) to induce LmACP19 recombinant protein with 0.2 mM isopropyl β-D-1-thiogalactopyranoside (IPTG), and the recombinant protein was purified using Ni-NTA affinity column as described by our previous methods [33]. Purified protein was detected using 12% (*w*/*v*) sodium dodecyl sulfate polyacrylamide gel electrophoresis (SDS-PAGE). Then, New Zealand White rabbits were injected to prepare polyclonal antibodies.

To localize the LmACP19 protein in wing pads from the middle to late instar stages, immune staining was performed as previously described [19]. In brief, paraffin sections (5 μm) of the wing pads from N5D3 and N5D7 were prepared for detection of the LmACP19 protein by incubation with the LmACP19 polyclonal antibodies (1:100) as a primary antibody at 4 °C overnight. After washing with phosphate buffered saline (PBS) three times for 5 min each, the tissues were then incubated with Cy3-Affinipure Donkey Anti-Rabbit (Jackson ImmunoResearch, West Grove, PA, USA) secondary antibody for 1 h at room temperature, followed by incubation with 1 mg/mL Fluorescent Brightener 28 (FB28) (Sigma, Louis, MO, USA) for 5 s to detect chitin [34]. SYTOX R Green nucleic acid stain (Life Technologies, Carlsbad, CA, USA) was added to label nuclei at a dilution of 1:50,000. The stained tissues were imaged using an LSM 880 confocal laser-scanning microscope (Zeiss, Oberkochen, Germany) at 60× magnification. All the images in each staining experiment were collected under exactly the same conditions.

### 4.5. RNA Interference (RNAi)

The forward and reverse primers (Supplementary Table S2) harboring T7 RNA polymerase promoter sequences were designed to synthesize double-stranded RNA (dsRNA) for *LmACP19* (ds*LmACP19*) and green fluorescent protein gene *GFP* (ds*GFP*, control) using a T7 RiboMAX™ Express RNAi System (Promega, Madison, WI, USA) as described previously [31]. The synthesized ds*GFP* or ds*LmACP19* was dissolved in appropriate volumes of deionized water to a concentration of 2.0 μg/μl, and their integrity was confirmed using 2% agarose gel analysis. Ten micrograms (5 μl) of ds*LmACP19* was injected into the hemocoel between the second and third abdominal segments of fourth-instar nymphs or fifth-instar nymphs by using a microsyringe. The same amounts of ds*GFP* were injected for the control. The relative transcript level of *LmACP19* was measured after injection of dsRNAs by RT-qPCR as described above. Four biological replicates were applied for ds*GFP* or ds*LmACP19* injections. The visible phenotype changes of remaining nymphs maintained under the same conditions as described above were recorded every day until the nymphs started to molt to the fifth-instar nymphs or adults.

### 4.6. Hematoxylin–Eosin Staining and Transmission Electron Microscope

To explore the effect of *LmACP19* RNAi on wing development, micro-sectioning and hematoxylin–eosin (H&E) staining was performed. Wing pads were dissected from day 2 of fifth-instar nymphs after injection of ds*GFP* or ds*LmACP19* in fourth-instar nymphs. In brief, paraffin sections (5 μm) of the wing pads were prepared after fixing, dehydrating, and embedding, and then stained with the H&E as described previously [35]. The slides were viewed under an OLYMPUSBX51 microscope and photographed using an OLYMPUS digital camera.

To further examine the wing development from the ultrastructural level, the cuticular structure of wing was analyzed at day 2 of adults treated with dsRNA in fifth-instar nymphs by transmission electron microscope (TEM) as the previously described method [36]. The images were captured with JEM-1200EX transmission electron microscope (JEOL, Tokyo, Japan).

### 4.7. TdT-Mediated dUTP Nick-End Labeling (TUNEL) and Caspase Activity

Paraffin sections of the wing pads after dsRNA injection in fourth-instar nymphs were prepared as described in the section of H&E staining. DNA fragmentation in wing pads was detected using the TdT-mediated dUTP nick-end labeling (TUNEL) kit (Beyotime, Shanghai, China) according to the manufacturer’s instruction. The stained sections were imaged using an LSM 880 confocal laser-scanning microscope (Zeiss, Oberkochen, Germany) at 60× magnification. All images were collected under the same conditions.

For caspase activity, the caspase-3, caspase-8, and caspase-9 activity kits were used to determine their respective activity in the wing pads of fifth-instar nymphs after injection of ds*GFP* or ds*LmACP19* according to the manufacturer’s instructions (Beyotime, Shanghai, China).

### 4.8. Staining of Cytoskeleton and Caspase-3 by Immunohistochemistry

The paraffin sections of wing pads treated with ds*GFP* or ds*LmACP19* were prepared as described above for detecting the cytoskeleton and cleaved caspase-3 by immunohistochemistry. The samples were blocked in PBS containing 5% bovine serum albumin (BSA) and 0.1% Triton-X (PBST) for 1 h, followed by incubation with the tubulin or cleaved caspase-3 antibody (BBI, Shanghai, China) at a dilution of 1:100 at 4 °C overnight. After washing three times with PBST, the samples were incubated with Alexa FlourR 488 Rabbit IgG (Life Technologies, Carlsbad, CA, USA) as the secondary antibody (diluted 1:500) for 1 h at room temperature. For the cytoskeleton staining, the specimens were incubated with FB28 (Sigma, Louis, MO, USA) (1 mg/mL) for 5 s to detect chitin [34]. The nuclei were labeled using SYTOX R Green nucleic acid stain (Life Technologies, Carlsbad, CA, USA), and the images were captured as described in Section 4.4.

### 4.9. Statistical Analysis

The one-way analysis of variance in the SPSS software (IBM Corp., Armonk, NY, USA) was applied to analyze differences between different developmental stages and different tissues, followed by the Tukey’s test. The other data were analyzed statistically using an independent sample Student’s *t*-test. Significant differences are marked with asterisks (*, *p* < 0.05; **, *p* < 0.01; ***, *p* < 0.001).

## Data Availability

Data are contained within the article or Supplementary Materials.

## Supplementary Materials

Supporting information can be downloaded at: https://www.mdpi.com/article/10.3390/ijms23063106/s1.
