# Supplementary material for: Cuticle Protein LmACP19 Is Required for the Stability of Epidermal Cells in Wing Development and Morphogenesis of Locusta migratoria
Source: Int J Mol Sci. 2022 Mar 13;23(6):3106. doi: 10.3390/ijms23063106 (PMC8950940; doi:10.3390/ijms23063106)
Supplement: Supplementary file 1 [file ijms-23-03106-s001.zip › ijms-1622103-supplementary.pdf]

Supporting information

Cuticle protein LmACP19 is required for the stability of epidermal cells in wing development and morphogenesis of *Locusta migratoria*

Xiaoming Zhao[1#*], Ti Shao [1,2#], Yazhi Su[1,2], Jing Zhang[1,2], Xin Gou[1,2], Weimin Liu[1] and Jianzhen Zhang[1*]

[1]Institute of Applied Biology, [2]College of Life Science, Shanxi University, Taiyuan, Shanxi 030006, China

*Authors for correspondence: zxming@sxu.edu.cn (XM. Zhao), and zjz@sxu.edu.cn (JZ. Zhang)

[#]These authors contributed equally to this work

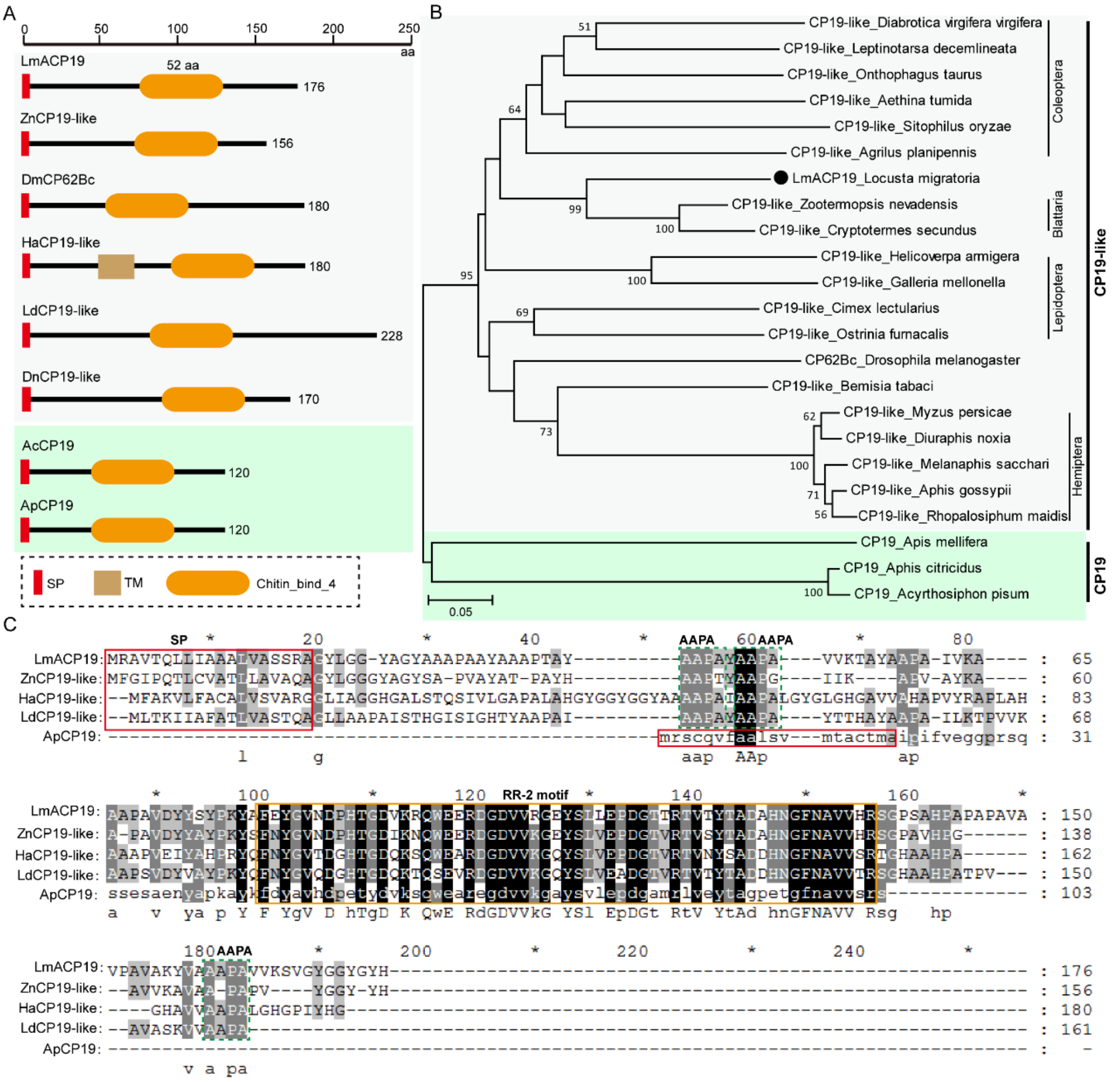

Supplementary Figure S1 The structure, phylogenetic tree and alignment of LmACP19 and its homology proteins

A. The structure of LmACP19 and its homologous proteins. B. Phylogenetic tree was constructed using LmACP19 and its homologous proteins. C. Alignment of LmACP19 and its homologous proteins. Red box indicates signal peptide, yellow box indicates RR-2 motif.

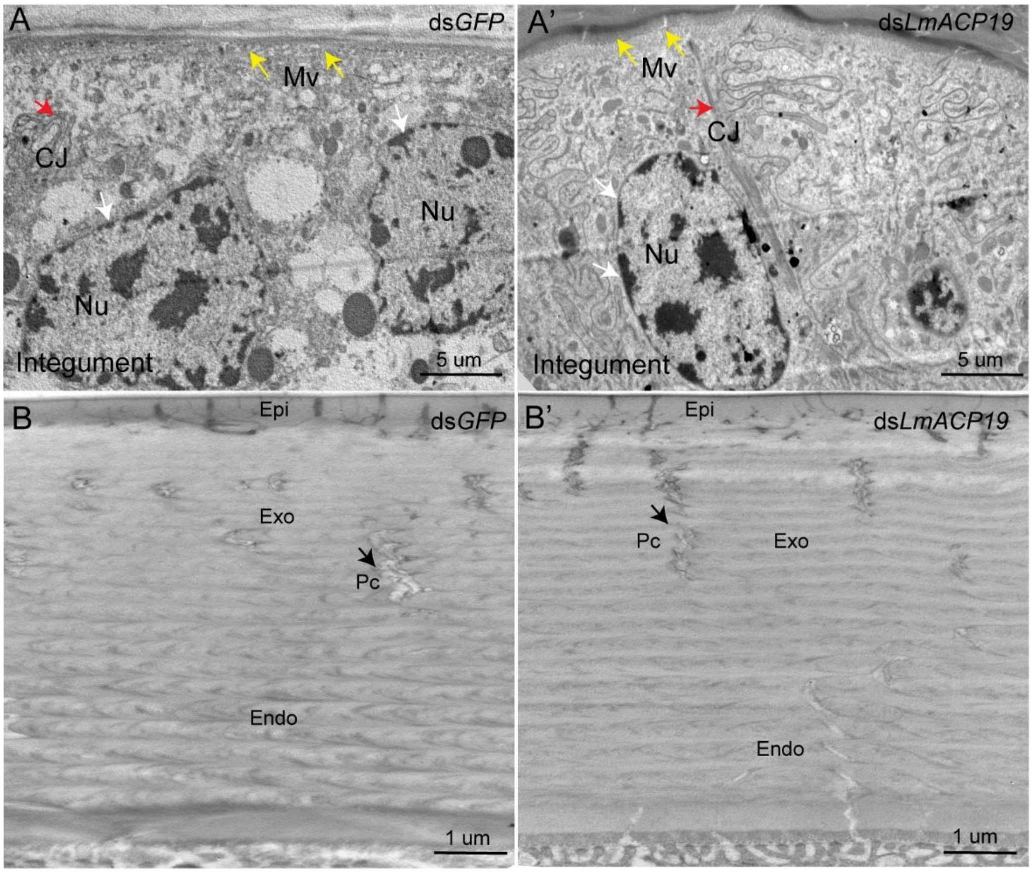

Supplementary Figure S2 Effect of ds*LmACP19* RNAi on structure of abdomen of *L. migratoria*

A-A'. The structure of epidermal cells of abdomen was observed after RNAi in fifth instar nymphs through TEM. Scale bar=5 μm. B-B'. The structure of abdomen cuticle was observed after RNAi in fifth instar nymphs through TEM. Epi: Epicuticle, Endo: Endocuticle, Exo: Exocuticle, Pc: pore canal, Nu: nucleus, Mv: microvilli, CJ: cell junction. Scale bar=1 μm. Black arrows indicate pore canal, white arrows indicate nucleus, yellow arrows indicate microvilli, red arrows indicate cell junction.

Supplementary Table S1 Species and GenBank accession number for phylogenetic tree used in this study.

Species	Protein name	GenBank accession number
Locusta migratoria	LmACP19	AMQ13160.1
Zootermopsis nevadensis	ZnCP19-like	XP_021930779.1
Agrilus planipennis	ApCP19-like	XP_018322294.1
Cryptotermes secundus	CsCP19-like	XP_023717583.1
Bemisia tabaci	BtCP19-like	XP_018897342.1
Aphis gossypii	AgCP19-like	XP_027852039.1
Myzus persicae	MpCP19-like	XP_022176116.1
Diabrotica virgifera virgifera	DvvCP19-like	XP_028147757.1
Leptinotarsa decemlineata	LdCP19-like	XP_023016039.1
Diuraphis noxia	DnCP19-like	XP_015378874.1
Melanaphis sacchari	MsCP19-like	XP_025193493.1
Rhopalosiphum maidis	RmCP19-like	XP_026821010.1
Cimex lectularius	ClCP19-like	XP_014255734.1
Sitophilus oryzae	SoCP19-like	XP_030747919.1
Aethina tumida	AtCP19-like	XP_019875897.1
Helicoverpa armigera	HaCP19-like	XP_021188428.1
Onthophagus taurus	OtCP19-like	XP_022903306.1
Galleria mellonella	GmCP19-like	XP_026751028.1
Ostrinia furnacalis	OfCP19-like	XP_028168293.1
Drosophila melanogaster	CP62Bc	NP_001261293.1
Apis mellifera	AmCP-19	GB48065
Aphis citricidus	AcCP-19	AVP39993.1
Acyrthosiphon pisum	ApCP-19	NP_001155504.1

Supplementary Table S2 Primer sequences used in this study.

Genes	Primer sequences (5′-3′)	Application
pET32-LmACP19	F: CGCGGATCCATGCTGGTGGCGTCCAGCCGCG<br>R: ATTTGCGGCCGCTCACGACTTGACGACGGCGGGTG	Recombinant protein construction
ds*GFP*	F: TAATACGACTCACTATAGGGTGGAGAGGGTGAAGG<br>R: TAATACGACTCACTATAGGGGGGCAGATTGTGTGGAC	dsRNA
ds*LmACP19*	F: TAATACGACTCACTATAGGGGTCAAGGCCGCCGCCCCAG<br>R: TAATACGACTCACTATAGGGGACGGCGTTGAAGCCGTTG	
RPL-32	F: ACTGGAAGTCTTGATGATGCAG<br>R: CTGAGCCCGTTCTACAATAGC	RT-qPCR
LmACP19	F: ACCGACGAGCAGACCAGCCA<br>R: GTAGCCTCCCAGGTAGCCAG	
LmACP7	F: GCCGCTGCCGCCCCCATTGA<br>R: GTCGCCCTCGCGCGTCTCGT	
Nedd2	F: GGTTACGGGATGTTGAGTGG<br>R: CCTTATGTGGAGGTGGAGAT	
ALG2	F: TCGCCGCTCAATCAAGTCAT<br>R: TAGCCTCCAGCCATAATAAA	
Arp	F: GGTTACTCCGATTTCCATA<br>R: AAACACTACTCAACGACCC	
Apaf1	F: ACAGAGGAAGGTGGAATAAC<br>R: TGGTAGATTTCCCACAGACA	
